# Imaging-Based Markers of Sarcopenia in Lower Limb Ischemia: A Systematic Review

**DOI:** 10.3390/jcm15156107

**Published:** 2026-08-06

**Authors:** Oliwia Grzelak, Ignacy Bobrowski, Joanna Halman, Michał Gniedziejko, Mariusz Siemiński, Jacek Wojciechowski

**Affiliations:** 1 Faculty of Medicine, Medical University of Gdańsk, 80-210 Gdańsk, Poland; 2 Department of Emergency Medicine, Medical University of Gdańsk, 80-210 Gdańsk, Polandsieminski@gumed.edu.pl (M.S.); 3 Clinical Emergency Department, University Clinical Centre, 80-952 Gdańsk, Poland; 4 Department of Vascular Surgery, University Clinical Centre, Medical University of Gdańsk, 80-210 Gdańsk, Poland

**Keywords:** sarcopenia, imaging-based marker, lower limb ischemia, peripheral arterial disease, revascularization, computed tomography, body composition, frailty, mortality, prognosis

## Abstract

**Background/Objectives**: Imaging-based markers of sarcopenia have emerged as potential indicators of frailty and predictors of clinical outcomes in patients with lower limb ischemia undergoing revascularization. However, the available evidence remains inconclusive. This systematic review aims to provide a comprehensive overview of current imaging-based approaches to sarcopenia assessment and to evaluate their relationship with clinical outcomes in this population. **Methods**: This systematic review was conducted in accordance with the Preferred Reporting Items for Systematic Reviews and Meta-Analyses (PRISMA) 2020 guidelines and prospectively registered in the International Prospective Register of Systematic Reviews (PROSPERO) database: ID CRD420251229527. A literature search was performed in PubMed and Scopus to identify studies published between 1 January 2020 and 1 August 2025. Studies assessing imaging-derived markers of sarcopenia in adults undergoing revascularization for lower limb ischemia were included. Due to heterogeneity in imaging methods, sarcopenia definitions and reported outcomes, a narrative synthesis was performed. **Results**: Nine studies involving 2563 patients were included in this review. Computed tomography (CT) was the predominant imaging modality used for sarcopenia assessment, with muscle measurements most frequently obtained at the level of the third lumbar vertebra (L3). Commonly reported imaging-derived markers included the skeletal muscle index, psoas muscle-based parameters and muscle radiodensity. Assessment strategies varied across studies, particularly with respect to measurement techniques, anatomical landmarks and the thresholds used to define sarcopenia. Most studies reported an association between imaging-derived low muscle mass and increased long-term mortality; however, independent associations after multivariable adjustment were less consistent. Findings regarding postoperative complications and limb-related outcomes were limited and inconsistent. **Conclusions**: Current evidence suggests that imaging-derived muscle markers may provide additional prognostic information regarding long-term mortality in patients undergoing revascularization for lower limb ischemia, while associations with postoperative complications and limb-related outcomes remain inconsistent. However, the small number of retrospective studies and substantial methodological heterogeneity limit their comparability, highlighting the need for standardization in future research.

## 1. Introduction

Lower limb ischemia primarily occurs in elderly patients with multiple comorbidities including advanced atherosclerosis, diabetes, and cardiac and renal disorders. The term lower limb ischemia is used throughout this review to refer to the symptomatic spectrum of peripheral arterial disease (PAD), ranging from intermittent claudication to chronic limb-threatening ischemia (CLTI), with substantial differences in disease severity, functional impairment, and prognosis. Despite continuous improvements in surgical and endovascular management, outcomes in this population remain unfavorable. Advanced age and poor general condition increase perioperative risk, and even technically successful revascularization does not always lead to clinical improvement. Therefore, identifying patients at higher risk of complications is essential for optimizing treatment. Conventional risk factors, however, do not fully capture patients’ physiological reserve, highlighting the need for additional prognostic markers.

Sarcopenia, defined as the loss of skeletal mass and quality [1], is an important component of frailty [2] and has gained recognition as a potential predictor of poor surgical outcomes [3,4]. Importantly, it can be assessed using imaging studies routinely performed in patients undergoing vascular evaluation.

Computed tomography (CT) is the most commonly used method, allowing for the simultaneous assessment of muscle mass and quality. Other imaging modalities such as magnetic resonance imaging (MRI), ultrasound and dual-energy X-ray absorptiometry (DEXA) can potentially be used [1].

Growing evidence suggests that imaging-defined low muscle mass is associated with postoperative complications, mortality and longer hospital stay in surgical patients [4,5,6,7,8,9,10,11,12]. However, existing studies are inconclusive, with differences in imaging methods, measurement techniques, diagnostic thresholds and patient populations, which limits comparability and clinical relevance [13]. Although imaging-derived muscle markers have been investigated in patients undergoing lower limb revascularization, methodological heterogeneity and inconsistent findings highlight the need for a comprehensive synthesis of the available evidence.

The aim of this systematic review is to evaluate the prognostic value of imaging-based markers of body composition in patients with lower limb ischemia by identifying relevant studies, describing imaging-based assessment methods, summarizing their association with clinical outcomes and assessing the quality of the available evidence.

## 2. Materials and Methods

### 2.1. Study Design

This systematic review was conducted in accordance with the PRISMA 2020 (Preferred Reporting Items for Systematic Reviews and Meta-Analyses) guidelines [14]. The completed PRISMA 2020 checklist and flow diagram are provided in the Supplementary Materials (Supplementary Material Files S2 and S3). The study protocol was prospectively registered in the International Prospective Register of Systematic Reviews (PROSPERO) database (ID CRD420251229527) [15].

### 2.2. Search Strategy

An extensive literature search was performed in PubMed and Scopus to identify studies published between 1 January 2020 and 1 August 2025. The search was limited to studies published between January 2020 and August 2025 to provide a contemporary synthesis of evidence on imaging-derived muscle markers in patients undergoing lower limb revascularization. Search terms included peripheral arterial disease; lower limb ischemia; chronic limb ischemia; critical limb ischemia (CLI); chronic limb-threatening ischemia; PAD, CLI and CLTI; sarcopenia; muscle mass; muscle wasting; skeletal muscle index; psoas muscle area; psoas muscle index; psoas density; muscle density; myosteatosis; radiodensity; attenuation values expressed in Hounsfield Units (HU), as well as imaging-related terms including computed tomography (CT), magnetic resonance imaging (MRI), ultrasound and dual-energy X-ray absorptiometry (DXA; also abbreviated as DEXA). The search was limited to original human studies published in English and excluded reviews, meta-analyses, case reports, editorials and letters. The full search strategy is available in the Supplementary Materials.

### 2.3. Eligibility Criteria

#### 2.3.1. Inclusion Criteria

- Original studies involving human participants (prospective or retrospective designs).
- Adult patients (≥18 years) diagnosed with lower limb ischemia undergoing surgical or endovascular revascularization.
- Assessment of body composition using imaging techniques (CT, MRI, ultrasound (US), DEXA).
- Reporting of any clinical outcomes in relation to imaging-defined sarcopenia (e.g., postoperative complications, mortality, hospital stay, functional outcomes).

#### 2.3.2. Exclusion Criteria

Studies were excluded if they:

- Were reviews, systematic reviews, meta-analyses, case reports, conference abstracts, editorials or letters;
- Involved animal or in vitro models;
- Assessed sarcopenia without imaging-based methods;
- Included mixed populations without separate analysis for patients with lower limb ischemia.

### 2.4. Study Selection

All identified records were imported into a reference management system where duplicates were removed. Titles and abstracts were independently screened by two reviewers to identify potentially eligible studies. Full-text articles were then assessed for inclusion based on predefined criteria. Any disagreements between reviewers were resolved through discussion or consultation with a third reviewer.

### 2.5. Data Extraction

Two reviewers independently extracted data using a standardized form. Collected data included study characteristics (author, year, country, digital object identifier (DOI)/ PubMed identifier (PMID)), patient demographics, procedural details, imaging modality, methods of imaging-based sarcopenia assessment (including anatomical landmarks and cut-off values) and reported clinical outcomes. When data were missing or unclear, the information was recorded as not reported, and no attempts were made to impute missing data.

### 2.6. Quality Assessment (NOS)

The methodological quality of included studies was assessed using the Newcastle–Ottawa Scale (NOS) for observational studies [16]. Studies were evaluated across three domains: selection, comparability and outcome assessment. Each study was assigned a score ranging from 0 to 9 points. Quality assessment was conducted independently by two reviewers (OG, IB), with disagreements resolved through discussion with a third reviewer (JH).

### 2.7. Outcomes

The primary outcomes were postoperative complications and all-cause mortality. Postoperative complications were defined as adverse events occurring within 30 days after surgical or endovascular revascularization, including but not limited to cardiovascular, respiratory, renal and infectious complications.

Secondary outcomes included length of hospital stay, intensive care unit stay, discharge disposition, readmission rates, reintervention rates, and functional outcomes, as reported by individual studies.

### 2.8. Data Synthesis

A narrative synthesis of the results was conducted. Due to heterogeneity in study design, patient populations, imaging methodologies and the definitions of imaging-derived sarcopenia, a quantitative meta-analysis was not performed. The findings were summarized with a focus on imaging techniques, the definitions of imaging-derived sarcopenia, and associations between imaging-based markers and clinical outcomes. Due to the substantial heterogeneity of the included studies, a narrative synthesis was considered the most appropriate approach. Consequently, reporting bias and certainty assessments were not performed.

## 3. Results

### 3.1. Study Selection

A total of 197 records were identified through database searching in PubMed and Scopus. After the removal of duplicates, 139 remained and were screened based on title and abstracts, resulting in the exclusion of 128 records.

**Figure 1:**
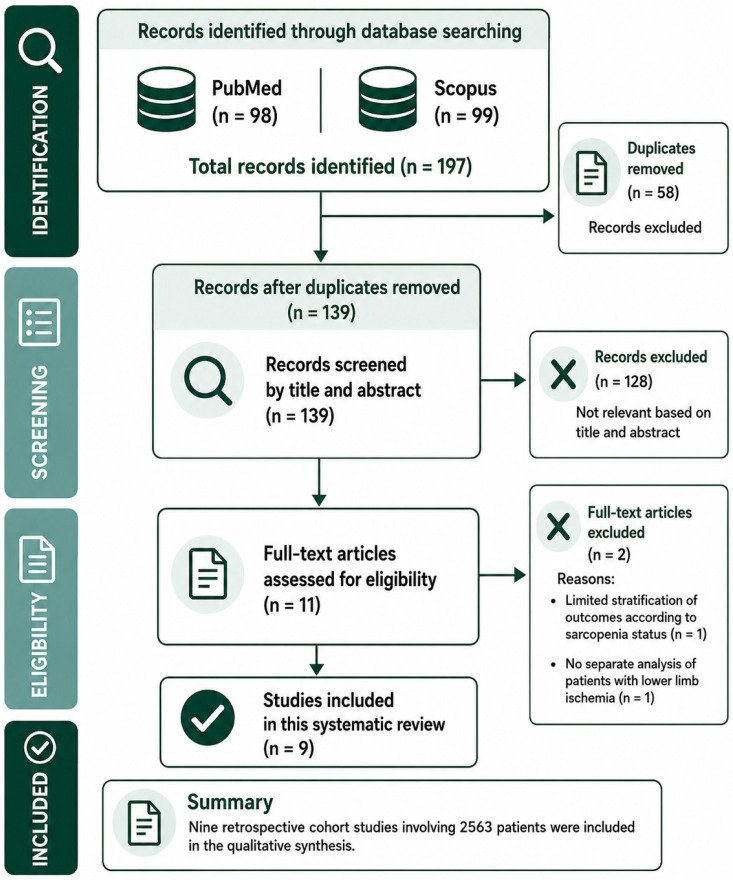
Flow chart of study selection process.

Eleven full-text articles were assessed for eligibility. Two full-text articles were excluded: one because outcomes were not stratified according to imaging-based sarcopenia status and one because patients with lower limb ischemia were not analyzed separately. Ultimately, nine studies were included in the qualitative synthesis (Figure 1).

### 3.2. Study Characteristics

Nine studies published between 2020 and 2025 were included, comprising a total of 2563 patients. Sample sizes ranged from 57 to 899 participants. All included studies were retrospective observational cohort studies, conducted in Europe and Asia.

The study population consisted of patients with lower limb ischemia, treated with either endovascular intervention or open surgical revascularization. Six out of nine studies focused exclusively on elective procedures [17,18,19,20,21,22], whereas two studies also included urgent cases [23,24], one of which additionally included emergency cases [24]. One study did not report the procedure setting [25]. The severity of lower limb ischemia was reported across studies using the Rutherford [17,19,21,23,25] or Fontaine classification [17,20]. Five studies included patients with advanced disease (Rutherford 3–6, 4–6, or 4–5 or Fontaine III-IV), while two studies included less advanced stages, and two did not specify disease severity (Table 1).

In all included studies, sarcopenia was evaluated using imaging performed prior to revascularization; muscle strength and physical performance were not assessed. CT was the predominant modality (eight out of nine studies) [17,18,19,20,21,22,23,25], one study used MRI [24], and ultrasound and DEXA were not used in any study.

### 3.3. Imaging Methods

CT was the predominant modality used for assessing imaging-defined sarcopenia in the included studies, typically performed at the level of the third lumbar vertebra (L3) [17,18,19,20,21,23]. In two studies, alternative anatomical landmarks were used, including the fourth lumbar vertebra (L4) level [25] and thigh muscle area [22]. MRI-based assessment was reported in one study, with measurement at the L4 level [24].

The most frequently reported imaging-derived markers included: the skeletal muscle index (SMI), psoas muscle area (PMA), psoas muscle index (PMI), skeletal muscle area (SMA), muscle radiodensity and myosteatosis.

The units used among studies varied depending on the marker being analyzed, including cm^2^, cm^2^/m^2^, mm^2^, and HU. Thresholds for imaging-based sarcopenia were highly heterogeneous and were mostly derived within each study. Eight studies reported explicit study-derived cut-off values for imaging-defined sarcopenia [17,18,19,20,21,22,23,25], whereas one study used the lowest quartile of the cohort distribution to define low muscle mass [24]; the threshold value of this quartile was considered the numerical cut-off value for imaging-defined sarcopenia for the purpose of this review. Assessment of muscle quality, such as radiodensity or myosteatosis, was reported in only three studies [18,23,25], while the majority of studies focused primarily on quantitative measures of muscle mass. The main characteristics of the included studies are presented in Table 1.

### 3.4. Outcomes

The detailed characteristics of the assessed outcomes, including imaging-based markers of sarcopenia, endpoints, and reported effect estimates, are summarized in Table 2.

#### 3.4.1. Mortality

Mortality was the most consistently assessed outcome, reported in seven of the nine included studies, using different endpoints such as overall survival, long-term all-cause mortality, all-cause mortality, and 2- or 3-year overall survival. All seven studies reported higher mortality among patients with imaging-derived low muscle mass in univariable analyses. However, after adjustment for potential confounders, four studies identified imaging-derived muscle markers as independent predictors of mortality in multivariable analyses [17,19,22,24], although in the study by Söderlund et al., this association was observed only in women [24]. Two studies found that the association was no longer statistically significant after adjustment [18,23], whereas one study did not perform multivariable analysis [25].

Cao et al. found that sarcopenic patients had a mortality risk more than twice as high as that of non-sarcopenic patients (HR 2.26, 95% CI 1.13–4.52), with significantly lower 3-year survival in the sarcopenic group (55.3% vs. 78.6%) [17].

Similarly, Morisaki et al. observed increased mortality in patients with imaging-derived low thigh muscle mass with 2-year survival rates of 55.1% in sarcopenic patients versus 86.5% in non-sarcopenic individuals (HR 2.64, 95% CI 1.11–6.7) [22].

Sivaharan et al. reported an even stronger association, showing notably worse overall survival (HR 5.8, 95% CI 1.8–19.1) in sarcopenic patients undergoing infra-inguinal surgical revascularization [19]. In multivariable analysis, skeletal muscle area remained independently associated with survival, with a 5% reduction in mortality risk per 1 cm^2^ increase in SMA (HR 0.95, 95% CI 0.92–0.99, *p* = 0.004).

In contrast, two studies did not confirm an independent association after adjustment for confounders.

Vedder et al. reported that while imaging-based sarcopenia was associated with increased mortality in univariable analysis (HR 2.82, 95% CI 2.05–3.86, *p* < 0.001), the association lost significance after adjustment for other prognostic factors (HR 1.40, 95% CI 0.97–2.01, *p* = 0.073) [18].

Halman et al. found that psoas-derived measures (PMA, PMD, PMI) were not independently associated with overall or late mortality, despite the fact that unadjusted mortality was higher among patients with lower muscle mass or density [23].

In women, higher psoas muscle area was independently associated with better survival (HR 0.71, 95% CI 0.60–0.90 per 1 SD increase in PMA), whereas no significant independent association was found in men [24].

Pereira-Neves et al. reported that reduced total psoas area was associated with significantly lower 1-year survival following revascularization (35.5% vs. 92.9%, *p* = 0.003) [25]. However, the study did not include multivariable regression analysis, limiting the ability to determine whether reduced psoas area was an independent predictor of survival.

Overall, although all seven studies suggested an association between imaging-derived low muscle mass and poorer survival [17,18,19,22,23,24,25], evidence for an independent prognostic effect was less consistent. Four studies demonstrated an independent association after multivariable adjustment [17,19,22,24], although the association reported by Söderlund et al. was limited to women [24]. Two studies reported a loss of significance after adjustment [18,23], and one study did not provide an adjusted analysis [25]. These findings suggest that imaging-derived markers of sarcopenia may have prognostic value; however, their independent contribution beyond established clinical risk factors requires further validation.

#### 3.4.2. Major Adverse Cardiovascular and Limb Events

Major adverse cardiovascular and limb-related events were reported less consistently across studies; however some demonstrated increased postoperative morbidity among sarcopenic patients.

Selçuk et al. reported that sarcopenic patients experienced significantly higher rates of early major adverse cardiovascular events (MACE) compared with non-sarcopenic individuals (9.8% vs. 0.7%, *p* = 0.002). Patients with imaging-derived low muscle mass had nearly twelve times greater odds of developing early postoperative MACE (OR 11.93). However, no significant association was observed for major adverse limb events (MALE) [20].

Similarly, Pereira-Neves et al. found that reduced total psoas area was significantly associated with major adverse cardiovascular and cerebrovascular events (MACCE) (*p* = 0.005), whereas psoas muscle density showed no significant association. The authors found no significant association between imaging-based sarcopenia and limb-related adverse events [25].

In contrast Halman et al. reported no independent relationship between psoas-derived measurements and either early or late postoperative complications. However, patients with lower psoas muscle density tended to experience more late complications, but statistical significance was not maintained in multivariable models [23].

Morisaki et al. reported no significant association between imaging-derived low thigh muscle mass and the risk of major amputation during 2-year follow-up [22], whereas Cao et al. similarly found that major amputation rates did not differ significantly between sarcopenic and non-sarcopenic patients (*p* = 0.897) [17].

These findings suggest that imaging-derived markers of sarcopenia appear to have stronger prognostic value for systemic cardiovascular risk than for limb-related complications. However, current evidence remains inconclusive due to the limited number of studies and heterogeneity in reported endpoints.

#### 3.4.3. Other Postoperative Complications

Postoperative complications other than MACE or MALE were reported by several included studies; however definitions and reporting standards considerably varied.

Halman et al. observed early postoperative complications in 15.8% of patients, including reoperation, wound infection, and in-hospital mortality. After adjustment for confounding variables, none of the assessed psoas-derived parameters showed an independent association with early postoperative complications [23].

Similarly, Dağlı et al. did not observe increased postoperative complication rates among sarcopenic patients undergoing endovascular treatment [21].

Although the study by Sivaharan et al. demonstrated significantly poorer survival among sarcopenic patients during follow-up, no statistically significant relationship was found between imaging-derived sarcopenia and procedure-related complications such as graft occlusion or wound infection [19].

Overall, available evidence regarding the relationship between imaging assessed low muscle mass and postoperative complications remains uncertain.

#### 3.4.4. Secondary Outcomes

Secondary outcomes were highly heterogeneous and were reported inconsistently across the included studies, which limited direct comparison between them.

Length of hospital stay was the most consistently reported secondary endpoint. Dağlı et al. demonstrated a significantly prolonged hospital stay in sarcopenic patients compared with non-sarcopenic patients (median 8 vs. 1 day, *p* < 0.001), indicating potentially slower postoperative recovery in patients with reduced muscle mass [21].

Additional postoperative outcomes were assessed only in individual studies and did not demonstrate consistent associations with imaging-derived sarcopenia, with prolonged hospital stay remaining the only relatively consistent secondary outcome across studies.

#### 3.4.5. Procedural Urgency

Two studies included patients undergoing urgent revascularization [23,24], while only one study additionally included emergency procedures [24]. However, neither study reported separate analyses according to procedural urgency. Consequently, the prognostic value of CT-derived sarcopenia markers in patients undergoing urgent or emergency revascularization could not be determined based on the available evidence.

### 3.5. Quality Assessment

The methodological quality of the included studies, assessed using the Newcastle–Ottawa Scale (NOS), ranged from moderate to high, with total scores between 6 and 9 points. The detailed scoring of each individual study is presented in Table 3.

All studies performed well in the selection domain, with clearly defined populations and an objective, imaging-based assessment of sarcopenia.

Greater variability was observed in the comparability domain. Even though several studies adjusted for important confounders including age, sex, and comorbidities, others provided limited or no adjustment, which may increase the risk of residual confounding.

Most studies used objective methods to assess outcomes and reported adequate follow-up. However, information on follow-up completeness was not always provided.

Overall, six studies were classified as high-quality (NOS ≥ 8), with the remaining three studies rated as moderate-quality (NOS 6–7). No studies were considered low-quality.

Common limitations included retrospective study design, single-center cohorts, heterogeneity in imaging-based sarcopenia definitions, a lack of standardized cut-off values, and inconsistent adjustment for confounders.

## 4. Discussion

### 4.1. Clinical Significance of Imaging-Derived Sarcopenia

Optimal patient selection remains one of the key challenges in vascular surgery, especially as the number of elderly patients with a high burden of comorbidities continues to rise. Although conventional risk stratification models take into account numerous factors, including age and comorbidities, they do not always accurately reflect an individual patient’s physiological reserve or their capacity to recover following surgery. For this reason, the imaging-based assessment of sarcopenia has gained increasing attention as a potential marker of frailty. This approach is particularly practical in vascular surgery where preoperative imaging is routinely obtained prior to revascularization, allowing us to assess low muscle mass without additional testing. Despite the increasing use of imaging-based body composition analysis, the field remains fragmented and methodologically inconsistent. Currently there is no standardized approach for incorporating imaging-based body composition measures into risk stratification in vascular surgery, and published studies vary considerably regarding the choice of imaging-derived markers, normalization approaches, the selected vertebral levels of measurements, imaging protocols and the methods used to define threshold values. As a result, inter-study comparability is limited, and the clinical relevance of these measures remains uncertain. The present review provides a comprehensive overview of current approaches to imaging-based sarcopenia assessment in vascular surgery, identifies important knowledge gaps and highlights priorities for future research.

### 4.2. Evidence from Other Surgical Specialties

The prognostic value of imaging-based sarcopenia assessment has been most extensively studied in oncologic surgery. Peng et al. showed that in patients undergoing pancreatic adenocarcinoma resection, sarcopenia assessed by the CT-derived skeletal muscle index (SMI) was independently associated with increased postoperative morbidity and reduced overall survival [3]. These findings were further supported by a systematic review and meta-analysis by Jones et al., which demonstrated that radiologically defined low muscle mass predicts higher postoperative morbidity and mortality following abdominal surgery [4]. Similar findings have been reported across various surgical populations, including oncologic, cardiovascular, emergency and transplant surgery. A meta-analysis by Knoedler et al. demonstrated that imaging-derived sarcopenia was associated with an increased risk of postoperative complications and mortality among patients undergoing surgery in these fields [5]. The negative impact of low muscle mass has also been demonstrated in cardiac surgery. Ansaripour et al. found that sarcopenic patients had higher mortality rates when undergoing cardiac procedures [6]. Taken together, evidence from other surgical specialties involving high-risk populations suggests that imaging-based markers of sarcopenia may hold promise as prognostic tools in patients with lower limb ischemia.

The association between imaging-based sarcopenia and adverse postoperative outcomes is supported by well-established biological mechanisms. As the primary reservoir of amino acids, skeletal muscle supports the acute-phase response, immune function and tissue repair during physiological stress. Consequently, reduced muscle mass limits metabolic reserve and the ability to withstand the catabolic response to surgery, increasing the risk of postoperative complications, delayed recovery and mortality [5,26].

### 4.3. The Main Findings of the Present Review

This review suggests that imaging-derived low muscle mass is associated with poorer long-term survival in patients undergoing lower limb revascularization. However, only some studies demonstrated an independent association after adjustment for potential confounders. In contrast, evidence regarding postoperative complications and limb-related outcomes remains inconsistent. This finding aligns with observations from other surgical specialties, where low muscle mass has shown stronger associations with long-term mortality than with procedure-specific complications [4,5,6]. These findings highlight the role of imaging-derived sarcopenia as a marker of reduced physiological reserve. Patients with impaired skeletal muscle status may have a limited capacity to respond to the metabolic demands of major interventions, tolerate postoperative stress and cope with the physiological demands of recovery. Because skeletal muscle serves as an important metabolic reserve, reduced muscle mass or quality may impair wound healing, recovery, and adaptation to postoperative complications, contributing to poorer long-term outcomes. Therefore, imaging-based body composition analysis may provide additional prognostic information by reflecting underlying biological frailty beyond conventional risk factors [17,19,22].

### 4.4. Imaging-Derived Markers of Sarcopenia

Of the nine included studies, six assessed only quantitative measures of skeletal muscle, including the SMI, SMA, PMI, PMA, and TPA, while three studies incorporated qualitative parameters alongside quantitative measures [18,23,25]. The parameters used to assess muscle quality were CT attenuation-based measures, including mean HU and PMD, both expressed in HU. Quantitative markers reflect muscle mass, whereas qualitative parameters provide information on muscle composition, including fatty infiltration and reduced muscle radiodensity, which may offer additional prognostic information [1,4]. Current evidence is insufficient to determine whether muscle quality provides superior prognostic value in patients with lower limb ischemia. The three studies incorporating quality-related parameters reported inconsistent findings. Heterogeneity in imaging markers, measurement sites and cut-off values limits direct comparison. Therefore, no single imaging-derived marker can currently be recommended as the preferred prognostic tool. Future studies directly comparing different imaging-derived markers within the same patient cohort may help identify the most clinically relevant parameter. Overall, quantitative markers, particularly the L3 skeletal muscle index and psoas muscle-based measurements, were the most frequently evaluated and showed the most consistent associations with mortality across the included studies. Evidence regarding qualitative markers, including muscle radiodensity and myosteatosis, remains limited because these parameters were assessed in only three studies [18,23,25], with inconsistent findings.

Although most studies reported an association between imaging-derived low muscle mass and mortality, independent associations after multivariable adjustment were less consistent. Moreover, the overall strength of the evidence remains limited by the observational design of all included studies and substantial methodological heterogeneity. Consequently, the current findings should be interpreted with caution.

### 4.5. Procedural Urgency

The available evidence regarding the prognostic value of imaging-derived sarcopenia markers in non-elective lower limb revascularization remains limited. Although two studies included patients undergoing urgent procedures [23,24], only one additionally enrolled patients treated in an emergency setting [24]. Neither study performed subgroup analyses according to procedural urgency. Therefore, the available evidence does not allow for conclusions regarding the prognostic value of imaging-based markers of sarcopenia according to procedural urgency. Future studies should investigate whether the predictive value of imaging-derived sarcopenia differs between elective, urgent and emergency lower limb revascularization.

### 4.6. Methodological Heterogeneity

Considerable methodological heterogeneity was observed across studies. Differences included imaging modality, anatomical landmarks, normalization methods, and diagnostic thresholds. Although CT at the L3 level was the most frequently used approach [17,18,19,20,21,23], measurements were also performed at L4 [24,25] and the thigh [22], and one study used MRI [24]. The lack of uniform criteria for defining imaging-derived sarcopenia, including differences in applied cut-off values, complicates comparison between cohorts and may contribute to variability in reported outcomes. In addition, the included studies evaluated heterogeneous patient populations, ranging from broader lower limb ischemia cohorts to patients with advanced chronic limb-threatening ischemia (CLTI) undergoing surgical or endovascular revascularization. It should also be noted that some studies used the older term “critical limb ischemia (CLI),” which has since been replaced by “chronic limb-threatening ischemia (CLTI)”. Both terms describe the same, most advanced stage of lower limb ischemia. Differences in disease severity, clinical presentation, and patient selection may also have influenced the prognostic value of imaging-derived muscle markers and contributed to the variability in the reported outcomes. The development of standardized definitions and measurement protocols for imaging-derived sarcopenia would improve comparability across studies and support a more consistent interpretation of future findings. Despite these limitations, the available evidence suggests that imaging-derived muscle markers may provide additional prognostic information to support perioperative clinical assessment.

### 4.7. Clinical Implications

Overall, no single imaging-derived biomarker consistently outperforms others across studies. The current findings suggest that the assessment of muscle status in vascular patients cannot be reduced to a single parameter but should be viewed as a combination of quantitative and qualitative features. Differences in study populations, methodologies and outcome reporting contribute to the variability in reported results. This reinforces the idea that sarcopenia is a complex, systemic condition linked to chronic inflammation, altered adipose tissue distribution and overall changes in body composition, rather than a simple reduction in muscle mass reflected by measurements of individual muscle size.

The findings of this review suggest that imaging-derived low muscle mass may help identify patients at increased risk of adverse outcomes. However, given the limited and heterogeneous evidence, they should currently be considered complementary prognostic markers rather than stand-alone tools for clinical decision-making. Careful monitoring may help identify adverse events at an early stage and allow for appropriate intervention without delay, potentially improving patient outcomes.

### 4.8. Future Perspectives

Since preoperative CT imaging is frequently available in patients undergoing evaluation for lower limb revascularization, automated or semi-automated body composition analysis may provide a feasible approach for identifying patients with reduced physiological reserve without increasing patient burden. Furukawa et al. emphasized that the assessment of frailty and sarcopenia may improve perioperative risk evaluation and support individualized management strategies in vascular surgery, including the optimization of nutritional status, rehabilitation and postoperative care planning [27]. In this context, imaging-derived sarcopenia assessment may also support shared decision-making by providing patients with a clearer understanding of expected risks and outcomes, thereby facilitating better-informed consent. Furthermore, recent advances in CT-based body composition assessment and artificial intelligence-assisted analysis may facilitate broader clinical adoption by improving reproducibility and reducing the time required for manual measurements [28].

However, before routine implementation, prospective multicenter studies with external validation are needed to establish standardized measurement protocols and clinically meaningful diagnostic thresholds and to determine whether incorporating imaging-derived body composition analysis into perioperative assessment provides additional prognostic value and improves clinical decision-making. Future studies should also directly compare different imaging-derived markers within the same patient cohorts to identify the most clinically relevant and reproducible assessment approach. Advances in artificial intelligence and automated segmentation may further improve reproducibility and facilitate broader clinical adoption [29,30,31].

In addition to CT and MRI, emerging imaging modalities such as photoacoustic imaging may further expand the assessment of tissue characteristics. By combining optical and ultrasound imaging, photoacoustic imaging provides information on tissue structure, vascularization, and optical absorption. Although its role in the assessment of skeletal muscle in patients with lower limb ischemia has not yet been established, it may represent an interesting direction for future research [32].

## 5. Conclusions

Imaging-based body composition analysis may complement existing clinical risk assessment in patients with lower limb ischemia undergoing revascularization.

Unlike conventional risk assessment, these markers appear to capture patients’ physiological reserve and frailty, which may explain their more consistent association with long-term mortality than with early postoperative outcomes.

Future research should prioritize standardized measurement protocols, clinically relevant diagnostic thresholds and prospective multicenter validation to determine whether imaging-derived muscle markers provide independent prognostic value and can be integrated into routine vascular care.

## 6. Limitations

This review had several limitations. The available evidence consisted of only nine studies that met the inclusion criteria. There was considerable heterogeneity across studies in terms of imaging-derived sarcopenia markers, measurement methodologies, cut-off values and reported outcomes, which limited their comparability. Additionally, studies included patients with different stages of lower limb ischemia that may have influenced clinical outcomes. As a result, a quantitative synthesis could not be performed, which should be considered in the interpretation of the findings. Limiting the search to studies published from 2020 onwards may have resulted in the exclusion of earlier relevant studies. However, this approach was chosen to provide a review focused on contemporary imaging-based assessment and recent evidence in patients undergoing lower limb revascularization.

## Figures and Tables

**Table 1:** Characteristics of included studies.

Author (Year)	Country	n	Lower Limb Ischemia Severity (Fontaine/Rutherford Classification)	Procedure Type (Endovascular/Open/Mixed)	Imaging Modality	Imaging-Based Marker of Sarcopenia	Marker Type	Cut-Off Value
Zhanjiang Cao (2023) [17]	China	137	Rutherford 4–6	Endovascular	CT	L3 SMI	Muscle quantity	SMI—Male: <40.8 cm^2^/m Female: <34.9 cm^2^/m^2^
Issi R. Vedder (2020) [18]	The Netherlands	686	Not reported	Mixed	CT	L3 SMI, muscle radiodensity (HU)	Muscle quantity + quality	SMI—Male: <41.2 cm^2^/m^2^ Female: <31.5 cm^2^/m^2^ Mean HU—BMI < 25 kg/m^2^: <33.6 HU BMI ≥ 25 kg/m^2^: <25.8 HU
Ashwin Sivaharan (2021) [19]	United Kingdom	116	Rutherford 3–6	Open	CT	L3 SMA	Muscle quantity	SMA—Male: <114 cm^2^ Female: <89.8 cm^2^
Nehir Selçuk (2023) [20]	Türkiye	217	Not reported	Mixed	CT	L3 PMI	Muscle quantity	PMI—Male: <5.5 cm^2^/m^2^ Female: <4.0 cm^2^/m^2^
Mustafa Dağlı (2024) [21]	Türkiye	100	Rutherford 2–5	Endovascular	CT	L3 SMA	Muscle quantity	SMA—Male: <114 cm^2^ Female: <89.8 cm^2^
Koichi Morisaki (2020) [22]	Japan	117	Fontaine III-IV	Mixed	CT	Standardized thigh muscle area	Muscle quantity	Standardized thigh muscle area: <9
Joanna Halman (2025) [23]	Poland	234	Rutherford 4–5	Mixed	CT	L3 PMA, PMI, PMD	Muscle quantity + quality	PMA: ≤8.87 cm^2^ PMI: ≤3.27 cm^2^/m^2^ PMD: ≤31.97 HU
António Pereira-Neves (2020) [25]	Portugal	57	Rutherford 3–6	Mixed	CT	L4 TPA, PMD	Muscle quantity + quality	TPA: <2175 mm^2^ PMD: <51.75 HU
Minea Söderlund (2025) [24]	Finland	899	Majority of patients were classified as Fontaine IIb-IV (790) with small proportion of Fontaine I-IIa (109)	Mixed	MRI	L4 PMA	Muscle quantity	PMA—Male: ≤6.2 cm^2^ Female: ≤4.5 cm^2^

Abbreviations: BMI—Body Mass Index; CT—Computed Tomography; HU—Hounsfield Units; L3—Third Lumbar Vertebra; L4—Fourth Lumbar Vertebra; MRI—Magnetic Resonance Imaging; SMI—Skeletal Muscle Index; SMA—Skeletal Muscle Area; PMI—Psoas Muscle Index; PMA—Psoas Muscle Area; PMD—Psoas Muscle Density; TPA—Total Psoas Area; n—Number of Patients.

**Table 2:** Study outcomes.

Author (Year)	Marker	Outcome	Statistical Values (Hazard Ratio/Odds Ratio/*p*-Value)	Main Finding
Zhanjiang Cao (2023) [17]	L3 SMI	3-year overall survival, major amputation	Mortality: HR 2.262 (95% CI: 1.132–4.518); *p* = 0.021; amputation: *p* = 0.897.	↑ Mortality risk; independent predictor; no association with amputation
Issi R. Vedder (2020) [18]	L3 SMI	Overall survival	Univariable HR 2.82 (95% CI 2.05–3.86), *p* < 0.001. Multivariable HR 1.40 (95% CI 0.97–2.01), *p* = 0.073.	Association lost after adjustment
Ashwin Sivaharan (2021) [19]	L3 SMA	Overall survival, major amputation; complications	Sarcopenia (defined by L3 SMA cut-off): HR 5.8 (95% CI 1.8–19.1), *p* = 0.001. Continuous SMA: HR 0.95/cm^2^, *p* = 0.004. Major amputation *p* = 0.046.	Worse survival; SMA independently prognostic; ↑ amputation risk
Nehir Selçuk (2023) [20]	L3 PMI	MACE, MALE	MACE: OR 11.925 (multivariable analysis), *p* = 0.002; MALE: NS.	↑ MACE risk; no association with MALE
Mustafa Dağlı (2024) [21]	L3 SMA	All-cause mortality, complications; length of stay	Mortality *p* < 0.001; LOS median 8 vs. 1 day, *p* < 0.001.	↑ Mortality and longer hospitalization
Koichi Morisaki (2020) [22]	Standardized thigh muscle area	2-year overall survival; major amputation	HR 2.64 (95% CI: 1.11–6.7); *p* = 0.03; amputation: NS.	Worse survival; no association with amputation
Joanna Halman (2025) [23]	L3 PMA, PMI, PMD	Early and late complications; mortality	All results *p* > 0.1 (multivariate model).	No independent association
António Pereira-Neves (2020) [25]	L4 TPA	All-cause mortality; MACCE; limb events	Mortality: *p* = 0.003; MACCE: *p* = 0.005.	↑ Mortality and MACCE risk; no limb association
Minea Söderlund (2025) [24]	L4 PMA	Long-term all-cause mortality	Females: HR 0.71 (95% CI 0.60–0.90) per 1 SD increase in PMA; *p* = 0.003.	Higher PMA independently associated with better survival in women; no significant association in men

Abbreviations: L3—Third Lumbar Vertebra; L4—Fourth Lumbar Vertebra; SMI—Skeletal Muscle Index; SMA—Skeletal Muscle Area; PMI—Psoas Muscle Index; PMA—Psoas Muscle Area; PMD—Psoas Muscle Density; TPA—Total Psoas Area; MACE—Major Adverse Cardiovascular Events; MALE—Major Adverse Limb Events; MACCE—Major Adverse Cardiovascular and Cerebrovascular Events; HR—Hazard Ratio; OR—Odds Ratio; CI—Confidence Interval; LOS—Length of Stay; NS—Not Significant.

**Table 3:** Quality assessment.

Author (Year)	Representativeness	Control from Same Population	Ascertainment of Exposure	Outcome Not Present at Start	Adjusted for Key Confounders	Outcome Assessment Method	Follow-Up Length Adequate	Follow-Up Completeness	NOS Total Score (0–9)	Quality Category
Zhanjiang Cao (2023) [17]	1	1	1	1	2	1	1	1	9	High
Issi R. Vedder (2020) [18]	1	1	1	1	2	1	1	1	9	High
Ashwin Sivaharan (2021) [19]	1	1	1	1	2	1	1	1	9	High
Nehir Selçuk (2023) [20]	1	1	1	1	1	1	1	0	7	Moderate
Mustafa Dağlı (2024) [21]	1	1	1	1	0	1	1	1	7	Moderate
Koichi Morisaki (2020) [22]	1	1	1	1	2	1	1	0	8	High
Joanna Halman (2025) [23]	1	1	1	1	2	1	1	0	8	High
António Pereira-Neves (2020) [25]	1	1	1	1	0	1	1	0	6	Moderate
Minea Söderlund (2025) [24]	1	1	1	1	2	1	1	1	9	High

Abbreviations: NOS—Newcastle–Ottawa Scale.

## Data Availability

No new data were created or analyzed in this study. Data sharing is not applicable to this article.

## Supplementary Materials

The following supporting information can be downloaded at https://www.mdpi.com/article/10.3390/jcm15156107/s1. File S1—Search strategy and study protocol; File S2—PRISMA 2020 checklist; File S3—PRISMA 2020 flow diagram.

## Abbreviations

The following abbreviations are used in this manuscript:

BMI	Body Mass Index
CI	Confidence Interval
CLI	Critical Limb Ischemia
CLTI	Chronic Limb-Threatening Ischemia
CT	Computed Tomography
DEXA	Dual-Energy X-ray Absorptiometry
DXA	Dual-Energy X-ray Absorptiometry
HR	Hazard Ratio
HU	Hounsfield Units
L3	Third Lumbar Vertebra
L4	Fourth Lumbar Vertebra
LOS	Length of Stay
MACE	Major Adverse Cardiovascular Events
MACCE	Major Adverse Cardiovascular and Cerebrovascular Events
MALE	Major Adverse Limb Events
MRI	Magnetic Resonance Imaging
NOS	Newcastle–Ottawa Scale
OR	Odds Ratio
PAD	Peripheral Arterial Disease
PMA	Psoas Muscle Area
PMD	Psoas Muscle Density
PMI	Psoas Muscle Index
PRISMA	Preferred Reporting Items for Systematic Reviews and Meta-Analyses
PROSPERO	International Prospective Register of Systematic Reviews
SMA	Skeletal Muscle Area
SMI	Skeletal Muscle Index
TPA	Total Psoas Area
US	Ultrasound

## References

[1] Cruz-Jentoft A.J., Bahat G., Bauer J., Boirie Y., Bruyère O., Cederholm T., Cooper C., Landi F., Rolland Y., Sayer A.A. (2019). Sarcopenia: Revised European consensus on definition and diagnosis. Age Ageing.

[2] Fried L.P., Tangen C.M., Walston J., Newman A.B., Hirsch C., Gottdiener J., Seeman T., Tracy R., Kop W.J., Burke G. (2001). Frailty in Older Adults: Evidence for a Phenotype. J. Gerontol. Ser. A Biol. Sci. Med. Sci..

[3] Peng P., Hyder O., Firoozmand A., Kneuertz P., Schulick R.D., Huang D., Makary M., Hirose K., Edil B., Choti M.A. (2012). Impact of Sarcopenia on Outcomes Following Resection of Pancreatic Adenocarcinoma. J. Gastrointest. Surg..

[4] Jones K., Gordon-Weeks A., Coleman C., Silva M. (2017). Radiologically Determined Sarcopenia Predicts Morbidity and Mortality Following Abdominal Surgery: A Systematic Review and Meta-Analysis. World J. Surg..

[5] Knoedler S., Schliermann R., Knoedler L., Wu M., Hansen F.J., Matar D.Y., Obed D., Vervoort D.M., Haug V., Hundeshagen G. (2023). Impact of sarcopenia on outcomes in surgical patients: A systematic review and meta-analysis. Int. J. Surg..

[6] Ansaripour A., Rad A.A., Koulouroudias M., Angouras D., Athanasiou T., Kourliouros A. (2023). Sarcopenia Adversely Affects Outcomes following Cardiac Surgery: A Systematic Review and Meta-Analysis. J. Clin. Med..

[7] Matsubara Y., Matsumoto T., Aoyagi Y., Tanaka S., Okadome J., Morisaki K., Shirabe K., Maehara Y. (2015). Sarcopenia is a prognostic factor for overall survival in patients with critical limb ischemia. J. Vasc. Surg..

[8] Weerink L.B.M., Van Der Hoorn A., Van Leeuwen B.L., De Bock G.H. (2020). Low skeletal muscle mass and postoperative morbidity in surgical oncology: A systematic review and meta-analysis. J. Cachexia Sarcopenia Muscle.

[9] Wang H., Yang R., Xu J., Fang K., Abdelrahim M., Chang L. (2021). Sarcopenia as a predictor of postoperative risk of complications, mortality and length of stay following gastrointestinal oncological surgery. Ann. R. Coll. Surg. Engl..

[10] Su H., Ruan J., Chen T., Lin E., Shi L. (2019). CT-assessed sarcopenia is a predictive factor for both long-term and short-term outcomes in gastrointestinal oncology patients: A systematic review and meta-analysis. Cancer Imaging.

[11] Cao Q., Xiong Y., Zhong Z., Ye Q. (2019). Computed Tomography-Assessed Sarcopenia Indexes Predict Major Complications following Surgery for Hepatopancreatobiliary Malignancy: A Meta-Analysis. Ann. Nutr. Metab..

[12] Kawaguchi Y., Hanaoka J., Ohshio Y., Okamoto K., Kaku R., Hayashi K., Shiratori T., Akazawa A. (2021). Does sarcopenia affect postoperative short- and long-term outcomes in patients with lung cancer?—A systematic review and meta-analysis. J. Thorac. Dis..

[13] Zumsteg D.M., Chu C.E., Midwinter M.J. (2020). Radiographic assessment of sarcopenia in the trauma setting: A systematic review. Trauma Surg. Acute Care Open.

[14] Page M.J., McKenzie J.E., Bossuyt P.M., Boutron I., Hoffmann T.C., Mulrow C.D., Shamseer L., Tetzlaff J.M., Akl E.A., Brennan S.E. (2021). The PRISMA 2020 statement: An updated guideline for reporting systematic reviews. BMJ.

[15] Grzelak O., Bobrowski I., Halman J., Siemiński M. (2025). Imaging-Based Markers of Sarcopenia in Lower Limb Ischemia: A Systematic Review [Internet]. PROSPERO. Report no.: CRD420251229527. https://www.crd.york.ac.uk/PROSPERO/view/CRD420251229527.

[16] Wells G.A., Shea B., O’Connell D., Peterson J., Welch V., Losos M., Tugwell P. (2011). The Newcastle–Ottawa Scale (NOS) for Assessing the Quality of Nonrandomised Studies in Meta-Analyses [Internet]. Ottawa Hospital Research Institute. http://www.ohri.ca/programs/clinical_epidemiology/oxford.asp.

[17] Cao Z., Zhao B., Jiang T., Zhang T., Yu X., Li Y., Wu W. (2023). Association of Sarcopenia With Mortality in Patients With Chronic Limb-Threatening Ischemia Undergoing Endovascular Revascularization. J. Surg. Res..

[18] Vedder I.R., Levolger S., Dierckx R.A.J.O., Zeebregts C.J., De Vries J.P.P.M., Viddeleer A.R., Bokkers R.P.H. (2020). Effect of muscle depletion on survival in peripheral arterial occlusive disease: Quality over quantity. J. Vasc. Surg..

[19] Sivaharan A., Boylan L., Witham M.D., Nandhra S. (2021). Sarcopenia in Patients Undergoing Lower Limb Bypass Surgery is Associated with Higher Mortality and Major Amputation Rates. Ann. Vasc. Surg..

[20] Selçuk N., Albeyoğlu Ş., Bastopcu M., Selçuk İ., Barutca H., Şahan H. (2023). Sarcopenia is a risk factor for major adverse cardiac events after surgical revascularization for critical limb ischemia. Vascular.

[21] Dağlı M., Gül E.B., Yiğit G., Gevrek M., Yılmaz M., Özen S., İşcan H.Z., Özen A. (2025). Sarcopenia is a possible risk factor for amputation after peripheral arterial interventions. Vascular.

[22] Morisaki K., Furuyama T., Matsubara Y., Inoue K., Kurose S., Yoshino S., Nakayama K., Yamashita S., Yoshiya K., Mori M. (2020). Thigh sarcopenia and hypoalbuminemia predict impaired overall survival after infrainguinal revascularization in patients with critical limb ischemia. Vascular.

[23] Halman J., Dybcio J., Myszczyński K., Kimilu N., Blacha A., Owedyk G., Wojciechowski J., Siemiński M. (2025). Limited Prognostic Value of Psoas Muscle Indices in Patients Undergoing Revascularization for Chronic Limb-Threatening Ischemia. Med. Sci..

[24] Söderlund M., Huhtamo H., Protto S., Hernesniemi J.A., Vakhitov D., Oksala N., Khan N. (2025). Magnetic Resonance Imaging—Derived Psoas Muscle Area and Survival in Patients Treated Invasively for Peripheral Arterial Disease. Scand. J. Surg..

[25] Pereira-Neves A. (2020). Impact of sarcopenia in aortoiliac occlusive disease in Mediterranean population. Turk. Gogus Kalp Dama.

[26] Wolfe R.R. (2006). The underappreciated role of muscle in health and disease. Am. J. Clin. Nutr..

[27] Furukawa H. (2022). Current Clinical Implications of Frailty and Sarcopenia in Vascular Surgery: A Comprehensive Review of the Literature and Consideration of Perioperative Management. Ann. Vasc. Dis..

[28] Pickhardt P.J. (2022). Value-added Opportunistic CT Screening: State of the Art. Radiology.

[29] Islam S., Kanavati F., Arain Z., Da Costa O.F., Crum W., Aboagye E.O., Rockall A. (2022). Fully automated deep-learning section-based muscle segmentation from CT images for sarcopenia assessment. Clin. Radiol..

[30] Urooj B., Ko Y., Na S., Kim I.O., Lee E.H., Cho S., Jeong H., Khang S., Lee J., Kim K.W. (2025). Implementation of Fully Automated AI-Integrated System for Body Composition Assessment on Computed Tomography for Opportunistic Sarcopenia Screening: Multicenter Prospective Study. JMIR Form. Res..

[31] Onishi S., Kuwahara T., Tajika M., Tanaka T., Yamada K., Shimizu M., Niwa Y., Yamaguchi R. (2025). Artificial intelligence for body composition assessment focusing on sarcopenia. Sci. Rep..

[32] Zhou J., Liang S., Park J.H., Nguyen A., Seong M. (2026). Burn depth assessment by photoacoustic imaging: A review. Methods.

