# Supplementary material for: Imaging-Based Markers of Sarcopenia in Lower Limb Ischemia: A Systematic Review
Source: J Clin Med. 2026 Aug 6;15(15):6107. doi: 10.3390/jcm15156107 (PMC13466395; doi:10.3390/jcm15156107)
Supplement: Supplementary file 1 [file jcm-15-06107-s001.zip › File S1 - search strategy and study protocol.pdf]

Imaging-Based Markers of Sarcopenia in Lower Limb Ischemia: A Systematic Review - STUDY PROTOCOL

1.Search strategy

PUBMED
("peripheral arterial disease" OR "lower limb ischemia" OR "chronic limb ischemia" OR "PAD" OR "critical limb ischemia" OR "critical limb threatening ischemia" OR "CLI" OR "CLTI")
AND
("sarcopenia" OR "muscle mass" OR "muscle wasting" OR "skeletal muscle index" OR "psoas muscle area" OR "psoas muscle index" OR "psoas density" OR "muscle density" OR "myosteatosis" OR "radiodensity" OR "attenuation" OR "Hounsfield")
AND
("computed tomography" OR "CT" OR "magnetic resonance" OR "MRI" OR "ultrasound" OR "DEXA" OR "DXA" OR "dual-energy x-ray absorptiometry")
NOT ("review"[pt] OR "systematic review"[ti] OR "meta-analysis"[ti] OR "case report"[ti] OR "editorial"[pt] OR "letter"[pt])
AND ("2020/01/01"[Date - Publication] : "3000"[Date - Publication])
AND ("english"[lang])

SCOPUS
TITLE-ABS-KEY("peripheral arterial disease" OR "lower limb ischemia" OR "chronic limb ischemia" OR "PAD" OR "critical limb ischemia" OR "critical limb threatening ischemia" OR "CLI" OR "CLTI")
AND
(TITLE-ABS-KEY("sarcopenia" OR "muscle mass" OR "muscle wasting" OR "skeletal muscle index" OR "psoas muscle area" OR "psoas muscle index" OR "psoas density" OR "muscle density" OR "myosteatosis" OR "radiodensity" OR "attenuation" OR "Hounsfield"))
AND
(TITLE-ABS-KEY("computed tomography" OR "CT" OR "magnetic resonance" OR "MRI" OR "ultrasound" OR "DEXA" OR "DXA" OR "dual-energy x-ray absorptiometry"))
AND PUBYEAR > 2019
AND (LIMIT-TO(LANGUAGE, "English"))
AND (LIMIT-TO(DOCTYPE, "ar"))

2. Inclusion criteria

- Original human studies (prospective or retrospective)

- Adult patients diagnosed with lower limb ischemia

- Assessment of sarcopenia or muscle quality using imaging techniques (CT, MRI, US, DEXA)

- Reporting of any clinical outcomes (e.g., postoperative complications, mortality, hospital stay, functional outcomes) in relation to sarcopenia

3. Exclusion criteria

Reviews, systematic reviews, meta-analyses, case reports, conference abstracts, editorials, letters

Animal or in vitro studies

Studies assessing sarcopenia without imaging

- Studies in mixed populations without separate analysis for patients with lower limb ischemia

4. PRISMA 2020 - Flow Diagram (for both databases: PubMed + Scopus)

Identification

Records identified: 197

Records identified from other sources (additional manual search): -

Total before deduplication: 197

Screening

Records after duplicates removed: 139

Records screened (title+abstract): 139

Records excluded (title+abstract): 128

Eligibility

Full-text articles assessed for eligibility: 11

Full-text articles excluded, with reasons: 2

- Lack of imaging-based sarcopenia assessment:-
- Absence of a lower limb ischemia population:-
- Non-original data (e.g., review article):-
- Other reasons:Lack of clear stratification of outcomes according to sarcopenia status in patients undergoing revascularization; No separate analysis of patients with lower limb ischemia with available CT measurements

Included

Studies included in qualitative synthesis (narrative systematic review):9

5. Quality assessment

The methodological quality of included studies was assessed using the Newcastle-Ottawa Scale (NOS) for observational studies. Each study was rated on selection, comparability, and outcome assessment domains. Disagreements were resolved through discussion.

Newcastle-Ottawa Scale (NOS) - Abbreviated Version (for Cohort / Case-Control Studies)

1. Selection (0–4 points)

Is the study population well defined and representative?

☐ Yes (1) ☐ No (0)

Does the control/comparison group (if applicable) originate from the same source population?

☐ Yes (1) ☐ No (0)

Are the diagnosis of lower limb ischemia and sarcopenia confirmed using objective criteria (e.g., imaging, validated cut-offs)?

☐ Yes (1) ☐ No (0)

Were participants free of the outcome of interest at baseline?

☐ Yes (1) ☐ No (0) ☐ N/A

2. Comparability (0–2 points)

Did the analysis adjust for key confounding variables (age, sex, BMI, comorbidities, ASA class)?

☐ Yes, ≥2 key variables (2)

☐ Yes, ≤1 key variable (1)

☐ No (0)

3. Outcome (0–3 points)

Were outcomes assessed independently / objectively (e.g., registries, adjudication committee)?

☐ Yes (1) ☐ No (0)

Was follow-up sufficiently long for the outcome to occur (≥30 days for complications, ≥1 year for survival)?
☐ Yes (1)　　☐ No (0)
Did ≥80% of participants complete follow-up, or were losses adequately accounted for in the analysis?
☐ Yes (1)　　☐ No (0)

6. Rationale
Lower limb ischemia often occurs in elderly patients affected by coexisting diseases, including advanced atherosclerosis, diabetes, cardiac and renal disorders. Unfortunately, advanced age, comorbidities and poor general condition of these patients frequently result in poorer surgical outcomes. Therefore, the best possible preoperative condition of the patient is the key to optimal treatment. In some cases even technically successful surgery fails to result in clinical improvement. For this reason, identifying the most burdened patients who are at the highest risk of complications has become crucial.
Sarcopenia-related frailty has gained attention as a potential predictor of adverse surgical outcomes, as loss of skeletal muscle mass can be assessed using imaging studies routinely performed in patients evaluated for vascular surgery. Computed tomography (CT) permits simultaneous assessment of muscle mass and quality using skeletal muscle index (SIM), muscle density in Hounsfield units and by evaluation of psoas muscle area. Alternative imaging modalities including magnetic resonance imaging (MRI), ultrasound and dual-energy X-ray absorptiometry (DEXA) can likewise be used to objectively quantify muscle characteristics.
Growing evidence suggests that sarcopenia assessed via imaging is associated with postoperative complications, mortality, and longer hospital stays in patients with lower limb ischemia. However, the studies are heterogeneous, which limits their comparability and practical application. Differences are mainly observed in imaging protocols, anatomical landmarks, measurement techniques, and diagnostic thresholds. Moreover, clarification is needed regarding the imaging marker to be used, and whether muscle mass, density, or a combination of these parameters provides the most reliable prognostic measure.

This systematic review will collect and critically assess original studies examining imaging-based markers of sarcopenia in patients with lower limb ischemia, in order to clarify their prognostic significance and potential role in risk stratification.
7. Objectives

The primary objective of this systematic review is to evaluate the prognostic value of imaging-based markers of sarcopenia in patients with lower limb ischemia.

Specific objectives are to:
1. Identify all original studies assessing sarcopenia or muscle quality in lower limb ischemia patients using imaging modalities such as CT, MRI, ultrasound or DEXA.
2. Describe measurement techniques, anatomical landmarks and diagnostic thresholds used to quantify muscle mass and density.
3. Summarize associations between imaging-derived markers and clinical outcomes, including complications, mortality, length of hospital stay and functional recovery.
4. Critically appraise methodological quality and highlight gaps in the literature.

8. PICO Framework

Population

Adults (≥18 years) diagnosed with lower limb ischemia, confirmed by imaging or surgical findings, who underwent assessment of muscle mass, muscle quality, or myosteatosis using imaging modalities (CT, MRI, ultrasound, or DEXA). Patients with chronic lower limb ischemia undergoing surgical or endovascular revascularization are eligible. Studies must report data specifically for patients with lower limb ischemia when mixed populations are included.

Intervention/Exposure

Assessment of sarcopenia, skeletal muscle mass, muscle quality, or myosteatosis using imaging modalities, including CT, MRI, ultrasound, or DEXA. Measurements may include psoas muscle area, skeletal muscle index (SMI), psoas muscle index (PMI), muscle cross-sectional area, or muscle radiodensity in Hounsfield units.

Outcomes

Primary outcomes: postoperative complications, all-cause mortality.

Postoperative complications occurring within 30 days after revascularization (open or endovascular). Acceptable measures include composite complication rates or individual complications (e.g., cardiac, respiratory, renal, infectious). Effect measures: odds ratio (OR), risk ratio (RR) or hazard ratio (HR) comparing patients with and without sarcopenia.
All-cause mortality: perioperative mortality (≤30 days) and long-term mortality as reported by each study. Effect measures: OR, RR, HR.

Secondary outcomes: length of hospital stay, length of ICU stay, discharge disposition, readmission rates, reintervention rates and functional recovery.

Length of hospital stay: the number of days from surgery to hospital discharge, reported as mean ± SD or median (IQR). Effect measure: mean difference or standardized mean difference between groups.
ICU length of stay: days from ICU admission to ICU discharge after revascularization. Effect measure: mean difference (MD) or standardized mean difference (SMD).
Discharge disposition: home vs facility (rehab/nursing home) at index discharge. Effect measure: odds ratio (OR) comparing sarcopenia vs no sarcopenia.
30-day readmission: any unplanned readmission within 30 days of discharge. Effect measure: OR or risk ratio (RR).
Reintervention/reoperation: any unplanned surgical or endovascular procedure related to the index revascularization during follow-up (report time window used by study; e.g., 30 days, 1 year). Effect measure: OR/RR; time-to-event as hazard ratio (HR) if reported.
Revascularization-related events: e.g., graft or stent failure, restenosis, reocclusion, distal embolization, wound complications, bypass graft stenosis/occlusion, or need for repeat reintervention, as defined by each study. Effect measure: OR/RR; HR for time-to-event.
Renal outcomes: acute kidney injury per study definition (prefer KDIGO when available) or new renal replacement therapy within 30 days. Effect measure: OR/RR.
Respiratory outcomes: pneumonia or prolonged ventilation (>48 h) within 30 days. Effect measure: OR/RR.

Transfusion requirement: perioperative packed red cell units transfused during index admission.Effect measure: MD/SMD.
Health-related quality of life/functional status: validated instruments (e.g., SF-36/12, EQ-5D) or mobility indices at the study's reported time points. Effect measure: MD/SMD.

9. Methods

A systematic literature search will be conducted in PubMed and Scopus, covering the period from 1 January 2020 to 1 August 2025. Search strategies will combine terms related to lower limb ischemia, sarcopenia, muscle mass/quality, and imaging modalities (CT, MRI, ultrasound, DEXA). Only original human studies in English will be included.

Study selection: Titles and abstracts will be screened independently by two reviewers. Full texts of potentially eligible studies will be assessed according to inclusion/exclusion criteria. Disagreements will be resolved by consensus or third-party adjudication.

Data extraction: Two reviewers will independently extract data on study characteristics, patient population, imaging methods, sarcopenia definitions, outcomes, and main findings.

Quality assessment: The Newcastle-Ottawa Scale (NOS) will be used to evaluate methodological quality.

Data synthesis: A qualitative synthesis will summarize methods and findings.

Registered in PROSPERO ID CRD420251229527
