# Supplementary material for: Imaging-Based Markers of Sarcopenia in Lower Limb Ischemia: A Systematic Review
Source: J Clin Med. 2026 Aug 6;15(15):6107. doi: 10.3390/jcm15156107 (PMC13466395; doi:10.3390/jcm15156107)
Supplement: Supplementary file 1 [file jcm-15-06107-s001.zip › File S3 - PRISMA 2020 flow diagram.pdf]

PRISMA 2020 flow diagram for new systematic reviews which included searches of databases and registers only

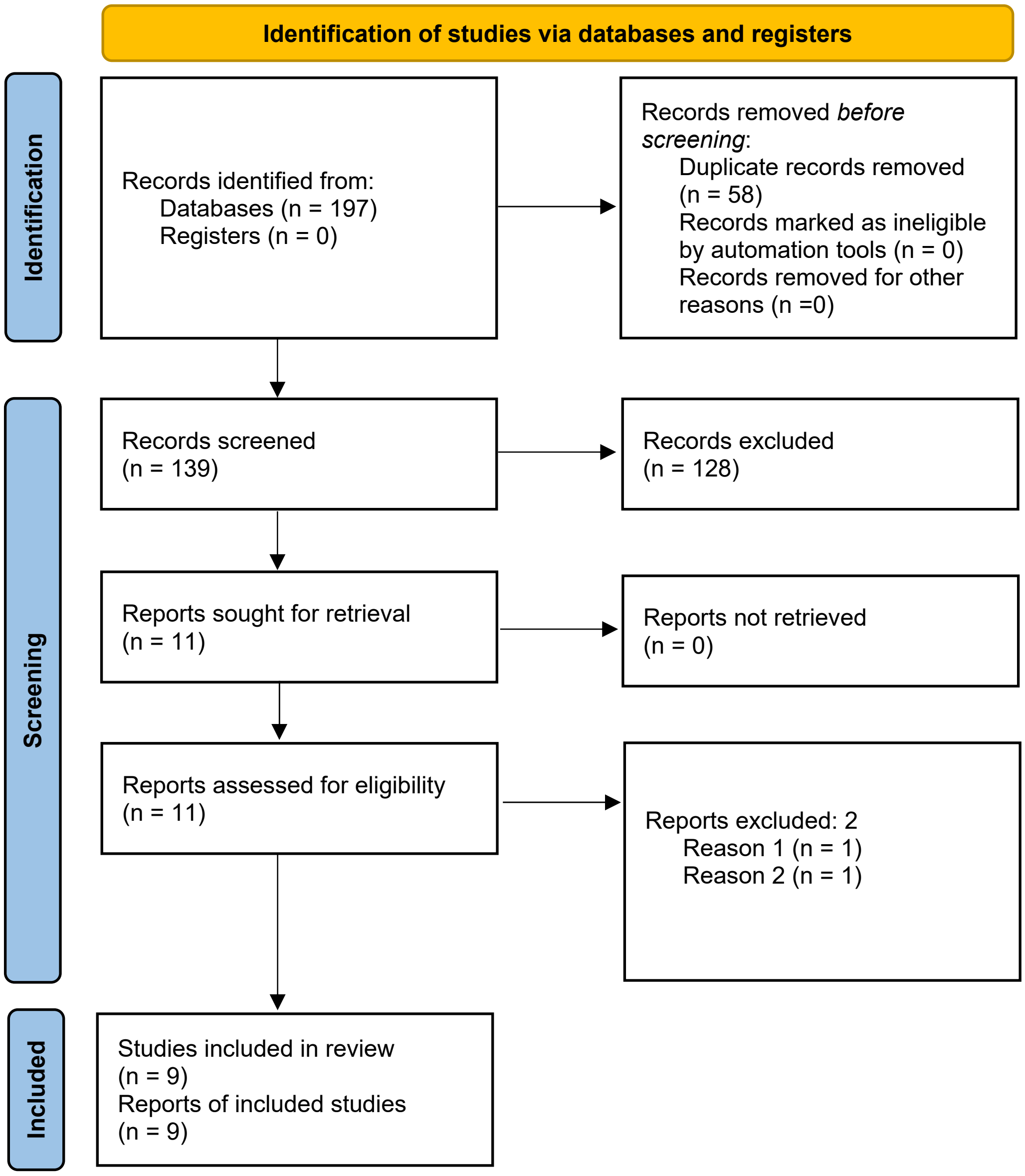

Reason 1: Limited stratification of outcomes according to sarcopenia status
Reason 2: No separate analysis of patients with lower limb ischemia

Source: Page MJ, et al. BMJ 2021;372:n71. doi: 10.1136/bmj.n71.

This work is licensed under CC BY 4.0. To view a copy of this license, visit https://creativecommons.org/licenses/by/4.0/
